# Low extracellular magnesium induces phenotypic and metabolic alterations in C2C12-derived myotubes

**DOI:** 10.1038/s41598-023-46543-9

**Published:** 2023-11-08

**Authors:** Monica Zocchi, Marco Bartolini, Jeanette A. Maier, Sara Castiglioni

**Affiliations:** Department of Biomedical and Clinical Sciences, Università di Milano, 20157 Milano, Italy

**Keywords:** Musculoskeletal models, Differentiation

## Abstract

Magnesium (Mg) has a pivotal role in upholding skeletal muscle health and optimizing performance. Its deficiency decreases muscle strength, and an association has been reported between Mg intake and sarcopenia. To gain a comprehensive understanding of the repercussions arising from low Mg concentrations on muscle behavior, we employed an in vitro model utilizing C2C12-derived myotubes. Myotubes cultured in low Mg show a significant reduction of thickness and a concomitant down-regulation of myosin heavy chain (MyHC), Myog and Myomixer. In parallel, myotubes shape their metabolism. Glycolysis is inhibited and beta-oxidation increases. These metabolic changes are consistent with the increase of MyHC I (slow) vs. MyHC II (fast) expression. We identified an essential player in these changes, namely nitric oxide (NO), as the increase in NO production appeared to orchestrate the observed modifications in myotube behavior and metabolism under low Mg conditions. Understanding these underlying mechanisms may pave the way for targeted interventions to ameliorate muscle-related conditions associated with Mg deficiency and contribute to enhancing overall muscle health and function.

## Introduction

Skeletal muscle is a highly plastic tissue which adapts its structure and metabolism in response to nutrients, contractile activity or mechanical load^1^. It consists of different types of fibers which are categorized as slow-twitch, which express type I isoform of myosin heavy chain (MyHC), and fast-twitch, which can be classified in type IIa (intermediate) or type IIb/x (the fastest), depending on the specific MyHC isoform expressed^2^. Slow-twitch fibers contribute to long-term endurance, while fast-twitch support power activities. While slow-twitch fibers use mitochondrial respiration, fast-twitch fibers have an anaerobic metabolism (type IIb/x) or use both (type IIa). At rest, muscle slow-twitch fibers produce ATP through beta-oxidation of fatty acids (FAs), generating Acetyl-CoA which enters the tricarboxylic acid (TCA) cycle. When an immediate energy production is necessary, the fast-twitch fibers (type IIa) uptake blood glucose through the mobilization of GLUT4 to the plasma membrane. Glucose is metabolized by glycolysis into pyruvate, which is then converted into Acetyl-CoA to be oxidized in the TCA cycle. When muscles have to support an intense exercise, the fast-twitch fibers (type IIb/x) also hydrolyze glycogen so that more glucose enters the glycolytic pathway. Under anaerobic conditions, pyruvate is converted into lactate. In response to modifications of functional demand, the muscles may undergo a phenotypic switch towards slow or fast fibers, resulting in a metabolic transition to oxidative respiration or glycolysis^3^.

Skeletal muscle fibers also adapt to nutritional challenges. Magnesium (Mg) is an essential element of life, because it regulates metabolism and homeostasis of all the tissues^4^. Besides being the activator of hundreds of enzymes, Mg binds many biological molecules, including proteins, RNAs, DNA, and ATP. In particular, ATP is active only when bound to Mg^2+^ (MgATP^2−^), because the linking of the cation allows the weakening of the terminal O-P of ATP, favoring the transfer of the phosphate^5^. Besides its role in energy production, in skeletal muscle cells Mg^2+^ is fundamental in regulating muscle contraction acting as a calcium (Ca^2+^) antagonist on Ca^2+^-permeable channels and Ca^2+^-binding proteins, such as troponin, thereby modulating myosin/F-actin interaction and the contraction of sarcomere^4^.

Since the Western diet is frequently characterized by a suboptimal Mg content^6^, the risk of chronic Mg deficiency is high. Indeed, hypomagnesemia can be considered one of the most underestimated electrolyte imbalance in the Western population^7^. An increased risk of low Mg status was also described in the elderly, and Mg deficiency is frequently associated with obesity, type 2 diabetes and metabolic syndrome^4^. In the muscle, Mg deficiency causes hyper-contractility, generating cramps, spasms, and weakness. A recent cross-sectional study has demonstrated a dose-dependent association between dietary Mg intake and sarcopenia^8^.

In a previous work^9^, we have investigated the effects of low extracellular Mg on myogenesis. Low Mg induces the production of reactive oxygen species (ROS) during the early phase of myoblasts’ differentiation. ROS directly inhibits myoblast membrane fusion, thus impairing myogenesis. In an in vivo model of mice fed a Mg deficient diet for 14 days, a slight decrease in body weight and in muscle Mg concentrations was found^10^. Although no variations in gastrocnemius muscle weight, fiber morphometry and capillarization were detected, TaqMan low-density array demonstrated that even a mild low Mg diet alters the expression of several genes involved in muscle regeneration, proteostasis, mitochondrial dynamics, excitation–contraction coupling and also in energy metabolism^10^. The expression of *Glut4*, which mediates glucose uptake into skeletal muscle cells, and *Citrate synthase*, which catalyses the first reaction of the Krebs cycle, is significantly reduced in mice fed a Mg deficient diet, demonstrating that myocytes metabolically adapt to the reduced availability of Mg. In the same experimental model, the downregulation of Perilipin *(PLIN)2* has been reported^10^. PLIN2 is a lipid droplet-associated protein and its expression mirrors the lipid content of the cells^11^, thus suggesting a potential dysregulation of lipid metabolism.

To characterize the events occurring in muscle cells under Mg deficiency, we used an in vitro model of murine myoblasts. Myoblasts were differentiated for 6 days into multinucleated myotubes, which were then cultured for 4 additional days in the presence of different extracellular Mg concentrations (Supplementary information, Fig. S1). Analysis on myotubes’ morphology, cell metabolism and MyHC isoform expression were performed to unveil the skeletal muscle cells responses to low extracellular Mg.

## Results

### Low extracellular Mg alters myotubes

**Figure 1:**
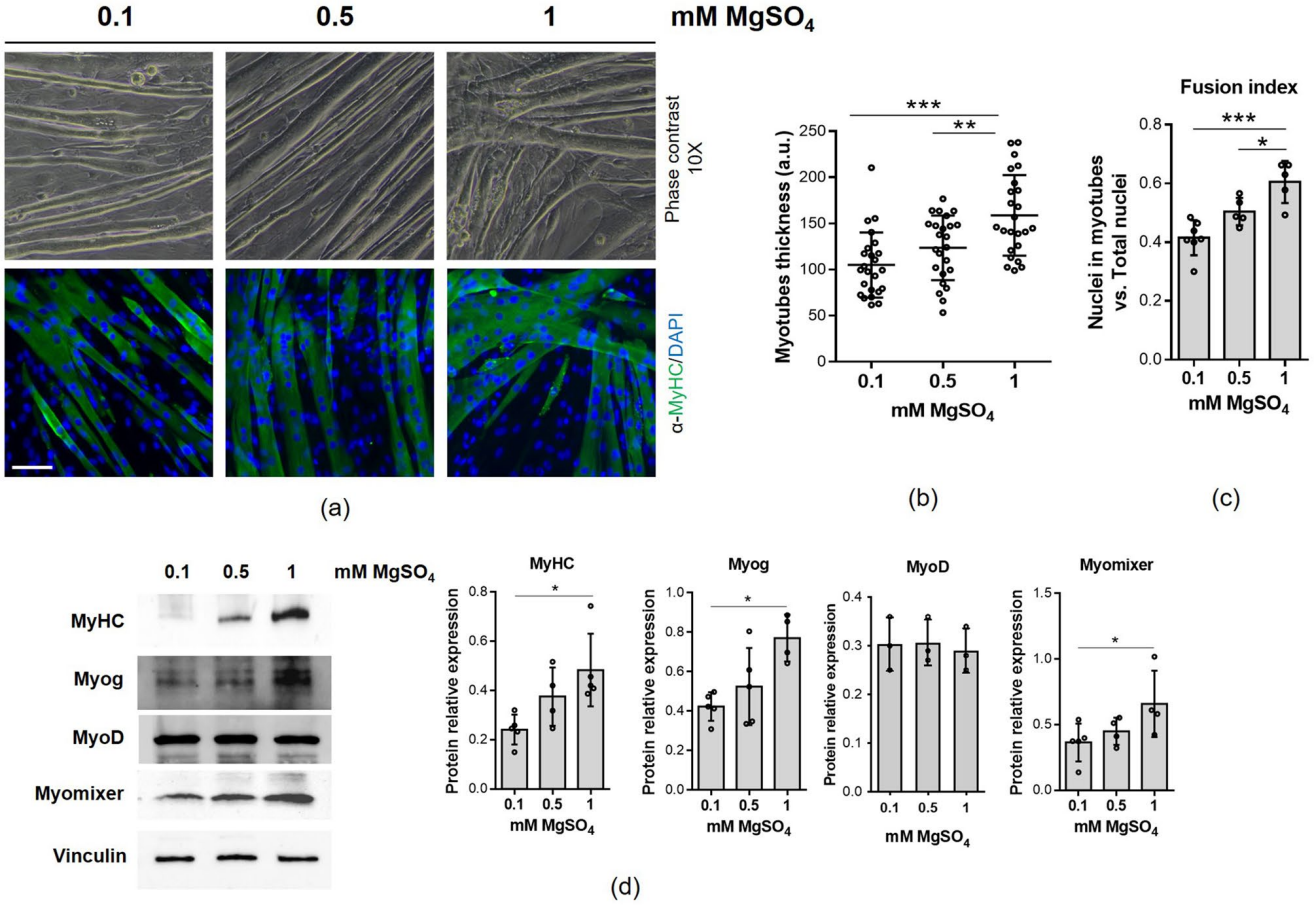
Myotubes cultured in low Mg show a reduced thickness compared to myotubes cultured in physiological Mg. Myoblasts were differentiated for 6 days and the obtained multinucleated myotubes were cultured for additional 4 days in the presence of 0.1 mM, 0.5 mM and 1 mM MgSO_4_. (**a**) Pictures were taken with an optical microscope (10 × magnification, upper panels). After immunofluorescence with antibodies against Myosin Heavy Chain (MyHC, green fluorescence), images were acquired using a fluorescence microscope (10 × magnification, lower panels). Nuclei were stained with 4’,6-diamidino-2-phenylindole (DAPI). (**b**) Myotubes thickness was obtained as the mean of three length measurements in three different positions of each myotube. Myotubes contained in at least 10 random images for each sample were measured. (**c**) Fusion index was calculated as the ratio of the number of nuclei within myotubes (> 2 nuclei) to the total number of nuclei in the field and quantified based on (**a**). (**d**) MyHC, Myog, MyoD and Myomixer expression were analyzed by western blot. Vinculin was used as control of loading. A representative blot (left) and densitometry performed on three independent experiments and obtained by ImageLab (right) are shown. *p ≤ 0.05; **p ≤ 0.01; ***p ≤ 0.001.

Myotubes obtained after 144 h of differentiation were cultured for 4 days in the presence of physiological Mg (1 mM MgSO_4_), or in mildly and severely Mg^2+^ deficient media (0.5 mM and 0.1 mM MgSO_4_, respectively). The myotubes were then analysed by optical microscope and by immunofluorescence using antibodies against the contractile protein Myosin Heavy Chain (MyHC). As shown in Fig. 1a, myotubes exposed to 0.5 mM and 0.1 mM extracellular MgSO_4_ show a significant reduction of their thickness and the fusion index values (nuclei in myotubes vs. total nuclei) when compared to cells in 1 mM MgSO_4_ (Fig. 1a–c). We then assessed the total amounts of MyHC, myogenin (Myog), a muscle-specific transcription factor essential for adult myofiber growth and muscle stem cell homeostasis^12^, MyoD, an early marker of myogenic commitment, and Myomixer, a key protein involved in myoblast membrane fusion^13^. Western blots demonstrate the reduction of MyHC, Myog and Myomixer levels (Fig. 1d) in cells cultured for 4 days in low Mg containing media. In the presence of a mild Mg deficiency, however, the difference did not reach statistical significance. The expression of the myogenic regulatory factor MyoD does not appear to be modulated in the different Mg conditions (Fig. 1d). The down-modulation of Myog and Myomixer is consistent with a decrease of the myotube regenerative capacity.

### Low extracellular Mg impairs glycolytic metabolism

Since Mg is a key regulator of hundreds of enzymes involved in metabolism, a low Mg status significantly impacts the metabolic profile of the cells. In particular, low intracellular Mg alters the tyrosine-kinase activity of the insulin receptor, resulting in post-receptor insulin resistance and impairing glucose utilization^14^.

**Figure 2:**
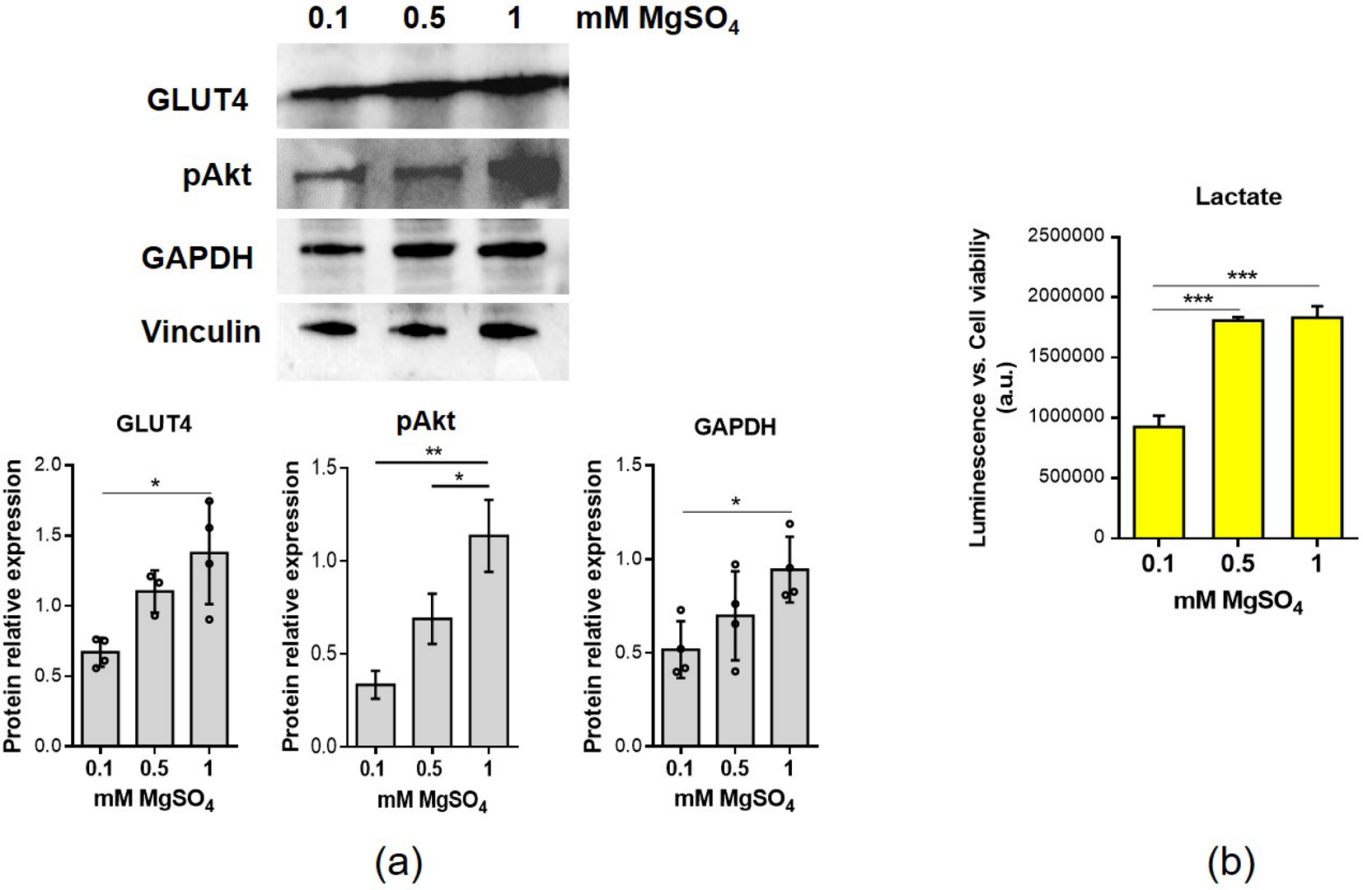
Low Mg affects glycolytic metabolism. Myotubes were cultured for 4 days in the presence of 0.1 mM, 0.5 mM and 1 mM MgSO_4_. (**a**) GLUT4, pAkt and GAPDH expression was analyzed by western blot. Vinculin was used as control of loading. A representative blot (upper panel) and densitometry performed on three independent experiments and obtained by ImageLab (lower panel) are shown. (**b**) Lactate production was measured and normalized on cell viability assessed by MTT assay. *p ≤ 0.05; **p ≤ 0.01; ***p ≤ 0.001.

On these bases, we investigated the effects of Mg deficiency on some aspects of glucose metabolism in myotubes. By western blot analysis, we show a reduction of the amounts of insulin-responsive glucose transporter GLUT4 in myotubes cultured in low Mg conditions vs. myotubes in physiological Mg (Fig. 2a). In low Mg conditions, we also detected a decrease of the amounts of pAkt, (Fig. 2a) which is involved in the downstream insulin receptor (INSR) signalling, regulating the GLUT4 mobilization to the plasma membrane^14^.

Moreover, Mg deficiency down-regulates GAPDH (glyceraldehyde-3-phosphate dehydrogenase), the sixth enzyme of the glycolytic pathway (Fig. 2a). Only in myotubes cultured in 0.1 mM MgSO_4_, we found a significant reduction of lactate production (Fig. 2b).

### Low extracellular Mg reduces the lipid droplets content and increases beta-oxidation

**Figure 3:**
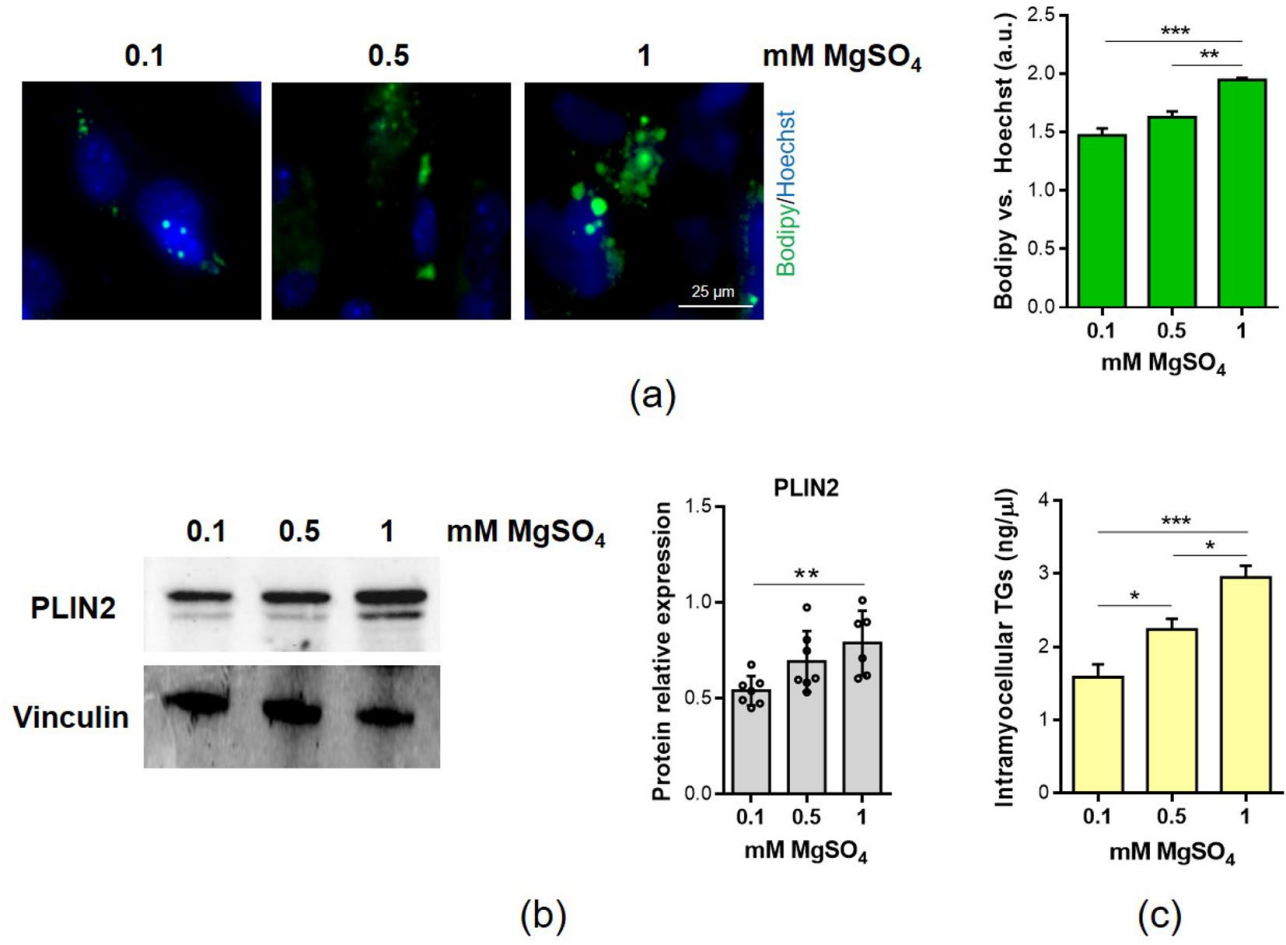
Low Mg reduces lipid droplets content. Myotubes were cultured for 4 days in the presence of 0.1 mM, 0.5 mM and 1 mM MgSO_4_. (**a**) Intracellular lipid droplets were stained with the neutral fluorescent probe Bodipy©. Nuclei were stained with Hoechst 33342. Images were acquired using a fluorescence microscope (10 × magnification, left panel). The fluorescence was acquired at Varioskan LUX Multimode Microplate Reader (right panel). (**b**) PLIN2 expression was analyzed by western blot. Vinculin was used as control of loading. A representative blot (left) and densitometry performed on three independent experiments and obtained by ImageLab (right) are shown. (**c**) Triglycerides (TGs) were quantified as described in methods. *p ≤ 0.05; **p ≤ 0.01; ***p ≤ 0.001.

We then focused on lipid metabolism. In myotubes cultured in Mg deficient media, we observed a significant decrease of lipid droplets content, as demonstrated by staining with the fluorescent probe Bodipy©, which detects intracellular neutral lipids (Fig. 3a). The reduction of lipid droplets content was further supported by the down-regulation of PLIN2, whose expression positively correlates with lipid droplets content in skeletal muscle^15^ (Fig. 3b). We also evaluated the amounts of triglycerides (TGs). As shown in Fig. 3c, the myotubes cultured in media containing low Mg show lower levels of TGs than controls.

**Figure 4:**
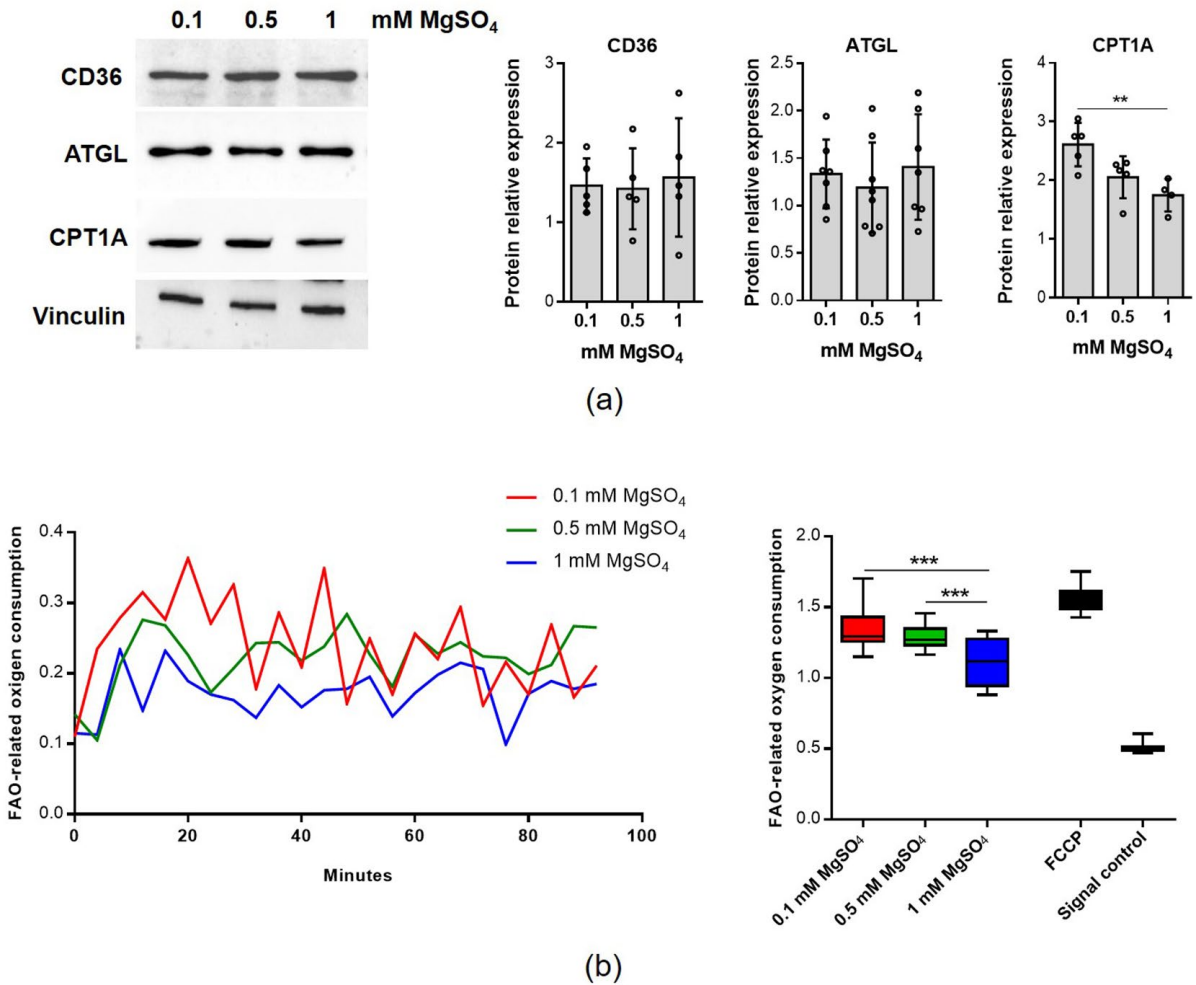
Low Mg increases fatty acid oxidation. Myotubes were cultured for 4 days in the presence of 0.1 mM, 0.5 mM and 1 mM MgSO_4_. (**a**) CD36, ATGL and CPT1A expression was analyzed by western blot. Vinculin was used as control of loading. A representative blot (left) and densitometry performed on three independent experiments and obtained by ImageLab (right) are shown. (**b**) Fatty acid oxidation (FAO) analysis was performed as described in methods. Treatment with the mitochondrial membrane potential uncoupler carbonyl cyanide-p-trifluoromethoxyphenylhydrazone (FCCP) was used as positive control. The results are both expressed as a time-course curve (left panel) and as a box plot graph (right panel). *p ≤ 0.05; **p ≤ 0.01; ***p ≤ 0.001.

To understand why lipid droplets content is reduced in low Mg, we analysed the total amounts of some molecules involved in lipid homeostasis by western blot. To investigate the uptake of fatty acids, we considered the transporter cluster of differentiation 36 (CD36)^15^. To get insights into TGs degradation, we analysed the lipid droplets-associated adipose triglyceride lipase (ATGL)^15^. We also studied the expression of the carnitine palmitoyl transferase 1 A (CPT1A), which is responsible for the uptake of fatty acids into the mitochondrial matrix^15^. While no differences emerged in the levels of ATGL and CD36, an increase of CPT1A was detected in cells cultured in Mg deficient media, even if a statistically significant upregulation is reached only in cells cultured in 0.1 mM MgSO_4_ (Fig. 4a). Coherently, a significant increase of oxygen consumption capacity related to fatty acid oxidation (FAO) was found in myotubes cultured in 0.1 and 0.5 mM MgSO_4_ (Fig. 4b). This finding might explain the reduction of lipid droplets content in myotubes cultured in low Mg.

### Low extracellular Mg decreases the amounts of fast MyHC

**Figure 5:**
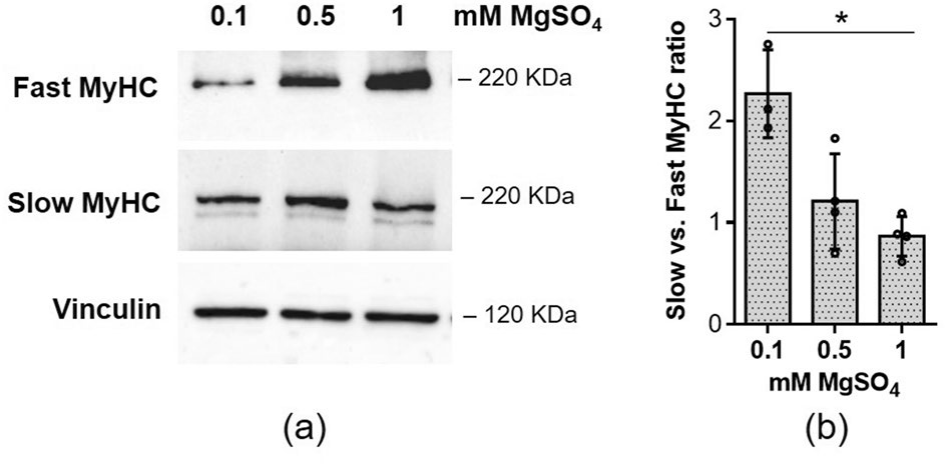
Low Mg decreases the amounts of fast MyHC. Myotubes were cultured for 4 days in the presence of 0.1 mM, 0.5 mM and 1 mM MgSO_4_. (**a**) Slow and fast MyHC isoform level was analysed by western blot. Vinculin was used as control of loading. A representative blot is shown. (**b**) The slow vs. fast ratio was calculated based on the densitometric analysis of the western blots. *p ≤ 0.05.

To assess whether alterations in the metabolic profile might serve as predictors for myotubes phenotypic characteristics, we analysed different MyHC isoforms by western blot (Fig. 5a). While MyHC I (slow) remained unaffected, a substantial down-regulation in the levels of MyHC II (fast) was evident when myotubes were cultured in Mg deficient media. Consequently, the ratio of MyHC I (slow) to MyHC II (fast) increased (Fig. 5b).

### Low extracellular Mg inhibits autophagy

**Figure 6:**
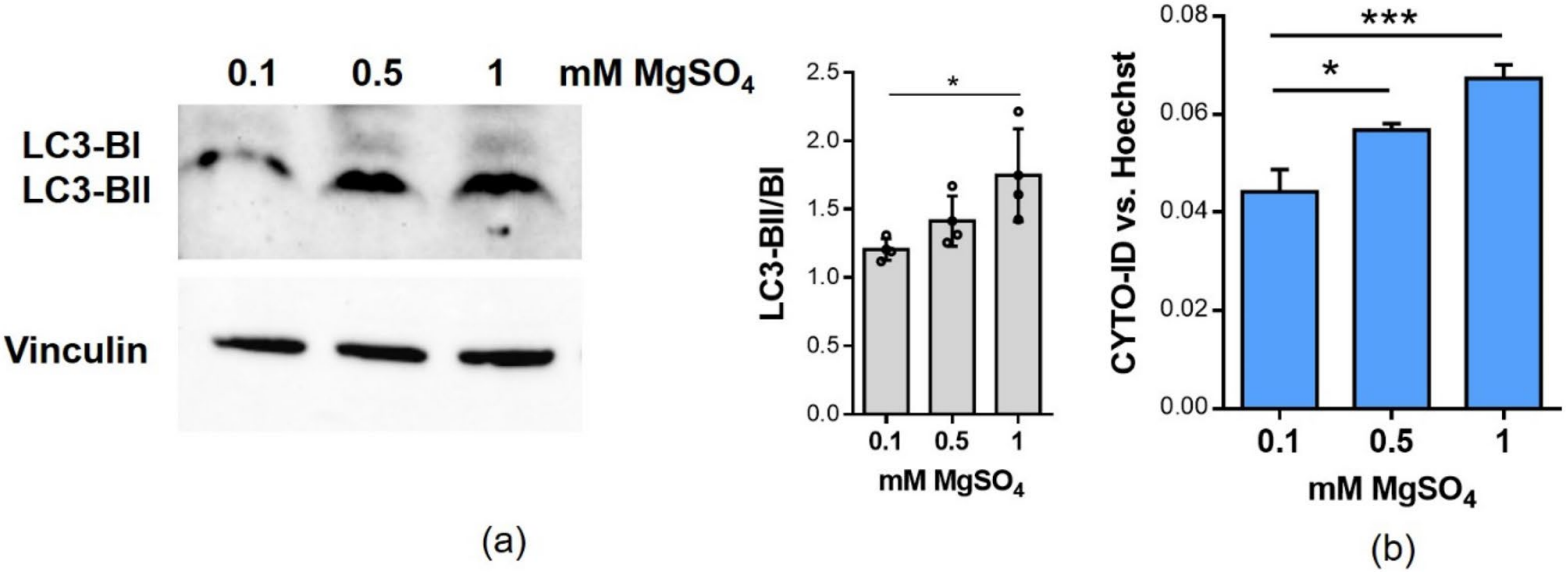
Low Mg affects autophagy. Myotubes were cultured for 4 days in the presence of 0.1 mM, 0.5 mM and 1 mM MgSO_4_. (**a**) LC3‐BII/BI levels was analyzed by western blot. Vinculin was used as control of loading. A representative blot (left) and densitometry performed on three independent experiments and obtained by ImageLab (right) are shown. (**b**) Autophagic flux analysis was performed with CYTO-ID probe and normalized on nuclei stained with Hoechst 33342. *p ≤ 0.05; ***p ≤ 0.001.

It is known that the autophagy‐lysosome system, together with anabolic processes, has a critical role in the maintenance of fiber homeostasis^16^. Since (i) lipid droplets are involved in the autophagic process, promoting autophagosome biogenesis^17^, and (ii) lipid droplets content is reduced in low Mg, we analysed the effects of Mg deficiency on autophagy in multinucleated myotubes. As shown in Fig. 6, in myotubes cultured in low Mg conditions, we demonstrate a decrease of the ratio LC3-BII/BI (Fig. 6a), and a reduction of the autophagic flux (Fig. 6b).

### Low Mg increases nitric oxide production

**Figure 7:**
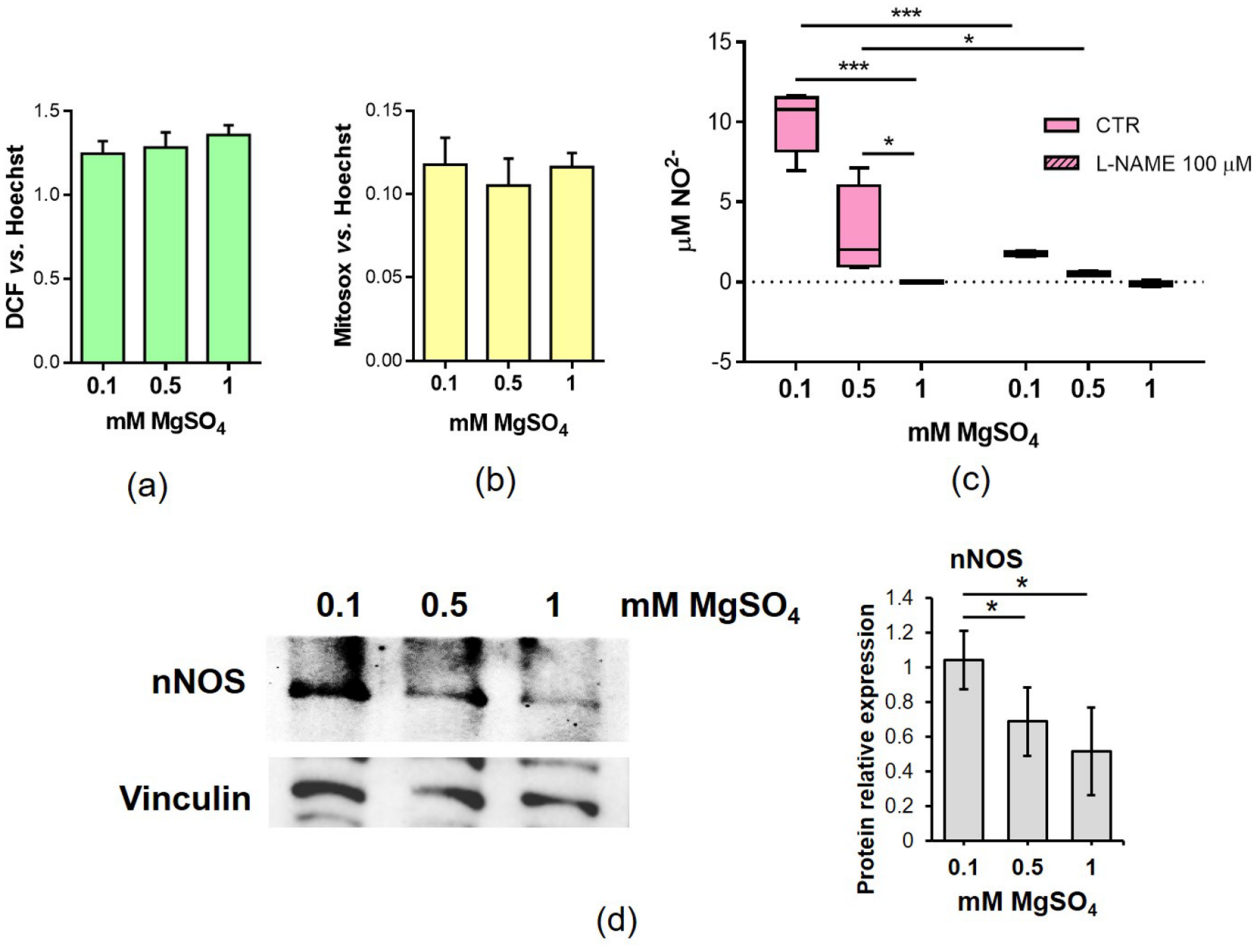
Low Mg increases NO production. Myotubes were cultured for 4 days in the presence of 0.1 mM, 0.5 mM and 1 mM MgSO_4_. (**a**) Total ROS accumulation was measured by 2ʹ,7ʹ-dichlorofluorescein (DCF) assay. Fluorescence was normalized on the nuclei stained with Hoechst 33342. (**b**) Mitochondrial ROS production was analysed with Mitosox and normalized on the nuclei stained with Hoechst 33342. (**c**) By Griess assay, nitrites were measured in the media of myotubes cultured in 0.1 mM, 0.5 mM and 1 mM MgSO_4_ in the presence or not of the nitric oxide synthases inhibitor L-N-Nitro arginine methyl ester (L-NAME). (**d**) Total amounts of nNOS was analyzed by western blot. Vinculin was used as control of loading. A representative blot (left) and densitometry performed on three independent experiments and obtained by ImageLab (right) are shown. *p ≤ 0.05; ***p ≤ 0.001.

To characterize the events occurring in myotubes in the presence of low Mg, we analysed two critical signalling molecules necessary for muscle homeostasis, i.e. reactive oxygen species (ROS) and nitric oxide (NO). ROS increase in low Mg has been shown to be directly involved in the impairment of myogenesis^18^. In myotubes cultured in low Mg conditions, we did not detect significant differences in total or mitochondrial-generated ROS (Fig. 7a,b). On the contrary, a significant increase of NO was measured (Fig. 7c). We then investigated the amounts of NO synthases (NOS) in our experimental model. While the inducible and endothelial isoforms of the enzyme were undetectable, neuronal NOS (nNOS) was upregulated under Mg deficient culture conditions (Fig. 7d).

### Nitric oxide mediates some of the effects of low Mg concentrations in myotubes

A role of NO in shaping skeletal muscle cell function is known^19,20^. We anticipated that the increased production of NO in myotubes cultured in low Mg might mediate the effects exerted by exposure to low Mg. We utilized the non-selective NOS inhibitor L-NAME, which prevents the NO increase in myotubes cultured in low Mg concentrations (Fig. 7c)^21^.

**Figure 8:**
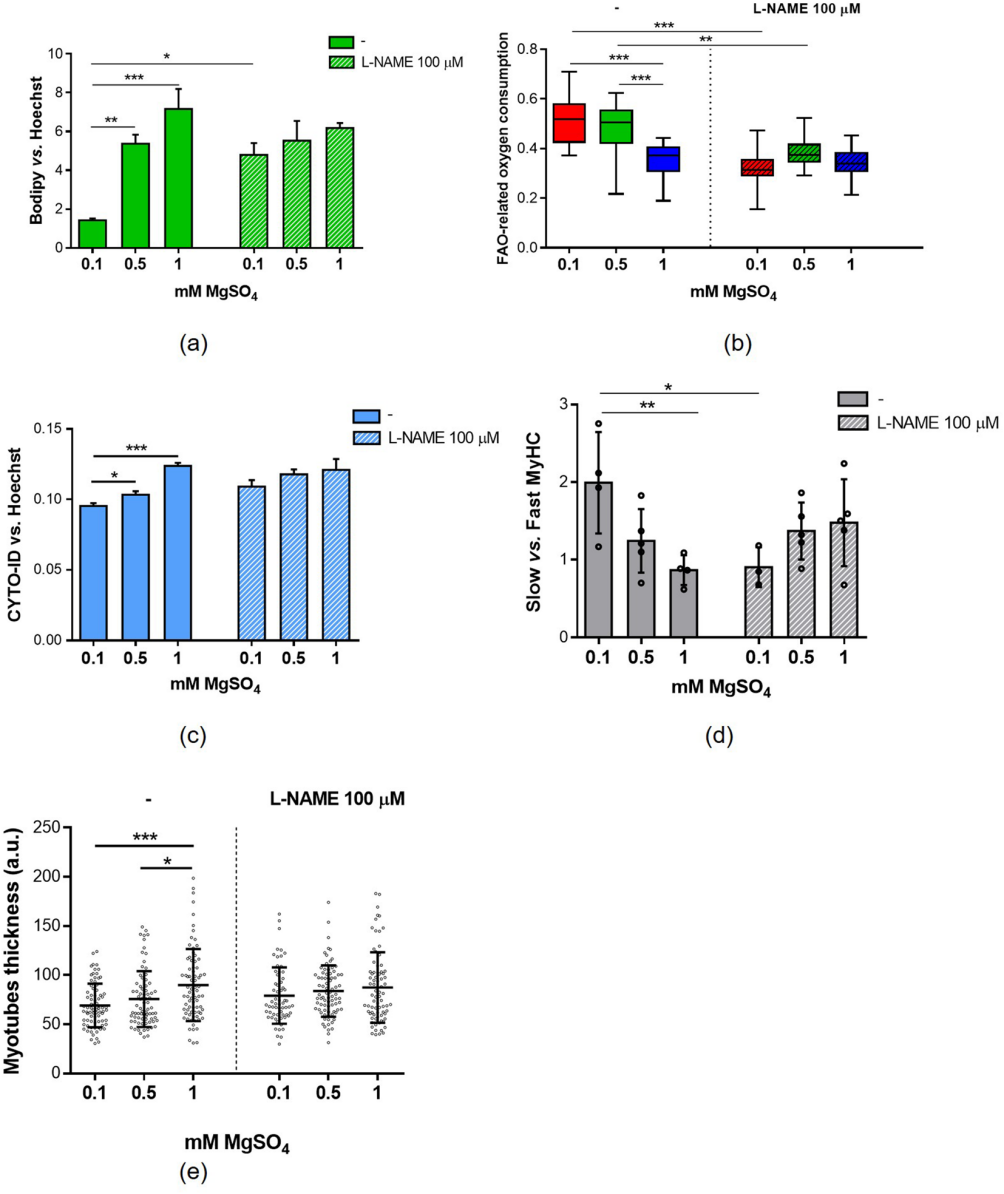
The inhibition of NO increase in low Mg prevents the fast-to-slow MyHC isoform switch. Myotubes were cultured for 4 days in 0.1 mM, 0.5 mM and 1 mM MgSO_4_ in the presence or not of the nitric oxide synthases inhibitor L-N-Nitro arginine methyl ester (L-NAME). (**a**) Intracellular lipid droplets were stained with Bodipy©. Nuclei were stained with Hoechst 33342. The fluorescence was acquired at Varioskan LUX Multimode Microplate Reader. (**b**) Fatty acid oxidation (FAO) analysis was performed as described in methods. (**c**) Autophagic flux analysis was performed with CYTO-ID fluorescent probe and normalized on nuclei stained with Hoechst 33342. (**d**) Slow and fast MyHC isoforms expression was analysed by western blot and the slow vs. fast ratio was calculated based on the densitometric analysis. The densitometric analysis performed on three independent experiments is shown. (**e**) Myotubes thickness was obtained as the mean of three length measurements in three different positions of each myotube. Myotubes contained in at least 10 random images for each sample were measured. *p ≤ 0.05; **p ≤ 0.01; ***p ≤ 0.001.

Culture for 4 days in low Mg media in the presence of L-NAME rescues lipid droplets content and beta-oxidation rate (Fig. 8a,b) to levels comparable to controls. Moreover, L-NAME prevents the inhibition of the autophagic flux in low Mg conditions (Fig. 8c) and restores MyHC I (slow) /MyHC II (fast) ratio in myotubes cultured in low Mg (Fig. 8d). Concerning myotube thickness, in the presence of L-NAME no significant differences emerged between myotubes cultured in low and physiological Mg (Fig. 8e).

## Discussion

Mg is essential for all living cells. Due to an insufficient dietary Mg intake and being associated with obesity, type 2 diabetes and metabolic syndrome, hypomagnesemia is a frequent condition and probably the most overlooked electrolyte imbalance in Western countries. The correlation between inadequate Mg intake and loss of skeletal muscle mass and function was demonstrated in different population cohorts^8,22,23^. In a murine model, a mild Mg deficiency suffices to alter the expression of genes critical for muscle physiology, including excitation–contraction coupling, muscle regeneration, mitochondrial dynamics and energy metabolism^10^. To get insights into the molecular basis of muscle impairment in response to low Mg, we used an in vitro model of myotubes obtained after myogenic differentiation of C2C12, which are then cultured in Mg deficient media for 4 days. Myotubes cultured in low Mg show a reduced thickness, down-regulate the contractile protein MyHC, the myogenic regulatory protein myogenin (Myog) and the fusogenic protein Myomixer. Surprisingly, low Mg does not affect the total amounts of MyoD, which is essential for activating the genetic program responsible for the transition from proliferative myoblasts to differentiating myotubes^12^. More studies are needed to understand this issue and also to investigate the modulation of the other members of the myogenic regulatory factors, such as MRF4 and Myf5.

It is known that Myog regulates the expression of genes required for myoblasts fusion, i.e. Myomaker and Myomixer^24^. During myogenesis, myoblasts fuse with each other to form multiple nuclear myotubes. In our previous work^18^ we have demonstrated that low extracellular Mg affects myogenesis by reducing Myomixer and impairing the myoblast fusion process. When myotubes are formed, a second phase of the fusion process occurs, in which myoblasts fuse with the myotubes to contribute to their growth^25^. On the bases of our data, we can speculate that the down-regulation of Myog in myotubes cultured in low Mg affects the second phase of the fusion process, resulting in the formation of thin myotubes. Since (i) we have previously demonstrated that autophagy inhibition is involved in the down-regulation of Myomixer expression, thereby impairing the fusion process^26^ and (2) we here show that autophagy is reduced also in myotubes, we hypothesize that the decline of the autophagic flux is involved in the impairment of muscle cell fusion upon exposure to low Mg concentrations, eventually through the down-modulation of Myomixer.

Skeletal muscle fibers adapt to nutritional challenges by modelling not only gene expression, but also metabolism and contractility. Initially, we analysed some aspects of glucose metabolism in response to low extracellular Mg concentrations. Glucose is transported from blood into the skeletal muscle cells through the glucose transporter type 4 (GLUT4). GLUT4 is an insulin-responsive protein, which is retained intracellularly and translocates to the plasma membrane in response to insulin^27^. It is known that low Mg induces insulin-resistance, because it inhibits the tyrosine-kinase activity of the INSR. As a consequence, the PI3K/Akt INSR-downstream pathway is affected leading to the decrease of GLUT4 mobilization to the membrane, and the reduction of glucose uptake and metabolism^14^. In myotubes cultured in low Mg we detected not only a significant reduction of GLUT4, but also a decrease of the activated Akt, which could impair INSR pathway, both leading to the decrease of glucose uptake by myotubes. In low Mg conditions we also detected the down-regulation of the glycolytic enzyme GAPDH which is involved in the oxidative phosphorylation of glyceraldehyde-3-phosphate in the presence of inorganic phosphate and nicotinamide adenine dinucleotide (NAD) into 1,3-diphosphoglycerate. In agreement with these in vitro results, GLUT4 and GAPDH are down-expressed in the gastrocnemius skeletal muscle of mice fed a low Mg diet for 14 days^10^. The pyruvate obtained from glycolysis can be converted into Acetyl-CoA and oxidized in the TCA cycle, or can be used to produce lactate through fermentation. In myotubes cultured in severe low Mg we demonstrate a reduction of the lactate production. The down-modulation of GAPDH and the reduced level of lactate suggests a reduction of glycolysis in low Mg. Lactate is known to modulate the function of skeletal muscle. C2C12-derived myotubes respond to the addition of lactate by increasing their size, as well as the diameter and the number of nuclei per fiber^28^. Accordingly, oral lactate administration increased murine muscle weight and fiber cross-sectional area^28^. On these bases, we suggest that the decreased amounts of lactate in myotubes cultured in low Mg medium contribute to the morphological alterations we observed in these cells.

Regarding lipid metabolism, fatty acids (FAs) uptake in muscle cells is mediated by CD36. Once inside the muscle cells, FAs follow different fates depending on the metabolic state of the cells. FAs can be immediately transported into the mitochondria for oxidation or, in resting conditions, can be stored in lipid droplets (LDs) as acyl-glycerides. LDs are vesicle-like organelles limited by a phospholipid monolayer which is associated with proteins belonging to the lipid droplet–associating protein family, among which PLIN2, which is highly expressed in skeletal muscle. Indeed, PLIN2 levels correlate with LD content and TG storage^15^. Since we found no differences in CD36 levels in cells cultured in different concentrations of extracellular Mg, it is feasible that low Mg does not impair FA entry in the cells. Nonetheless, neutral lipids and PLIN2 levels are significantly decreased, indicating a drop of LD content, associated with a decrease of intracellular TGs, in low Mg conditions. These data are in accordance with the results obtained in the gastrocnemius skeletal muscle of mice fed a Mg-deficient diet, in which the down-regulation of PLIN2^10^ and the reduction of TG content (Supplementary information, Fig. S2) occur. In all the Mg conditions tested, we did not find significant differences in adipose triglyceride lipase (ATGL) expression, the lipase which coats LDs and catalyzes TG lipolysis inducing the release of a single FA molecule. Interestingly, in cells cultured in low Mg media the enzyme carnitine palmitoyl transferase 1 A (CPT1A), which is involved in the FA uptake into the mitochondria, is upregulated and, in parallel, FA oxidation is enhanced. Therefore, it is likely that the increased transport of FAs to the mitochondria with the consequent gain in their beta-oxidation explains the reduction of the LD content caused by low Mg concentrations. Since LDs are critical in the autophagic process by promoting autophagosome biogenesis, it is not surprising that the autophagic flux is impaired in myotubes cultured in low Mg. Also in mice fed a low Mg diet, autophagy markers were down-regulated^10^. Recently, it was reported that high extracellular Mg activates autophagy via the AMPK/mTOR pathway in hepatocytes in vitro^29^, while it inhibits autophagy in chondrogenic cell line ATDC5 through the MAPK/ERK pathway^30^. The modulation of autophagy in response to different concentrations of Mg is still at the dawn. Considering that autophagy is a crucial mechanism for cellular homeostasis, it is likely that Mg concentrations differently impact the distinct cell types because of their peculiar function.

In skeletal muscle, different fiber types are characterized by different metabolic features, allowing the cells to rapidly adapt to different energy requests^31^. In particular, slow fiber metabolism is mainly based on mitochondrial respiration, which guarantees ATP generation for continuous muscle contractions for a long time. In fact, slow fibers contribute to long-term muscle endurance. Differently, fast fibers are characterized by a faster metabolism, based on aerobic and anaerobic glycolysis. This allows the fast fibers to rapidly respond to energy demand, to have a higher contraction rate and to generate a short burst of strength or speed. Moreover, muscle fibers can switch their features, in terms of both contractile protein expression and metabolism, and to calibrate the performance based on energy demands. For these reasons we investigated whether the aforementioned low Mg-dependent metabolic changes correlate with muscle fiber phenotypic adaptations. In myotubes cultured in low Mg we detected an increase of MyHC I (slow) vs. MyHC II (fast) expression ratio, thus advancing present understanding about the complex interplay between Mg availability, muscle fiber phenotype and metabolic dynamics.

We also brought to light a crucial player mediating these changes, i.e. NO. In myocytes cultured in low Mg, nNOS, the principal source of NO in skeletal muscle cells, is upregulated^19^ and determines NO accumulation. The increased NO production is instrumental in orchestrating the modifications in myotube behavior and metabolic responses under low Mg conditions. It is well established that NO assumes a pivotal role in the intricate regulation of skeletal muscle homeostasis^19^. Accordingly, perturbations in NO production compromise muscle fiber growth, leading to diminished muscular force and hindering the ability to sustain prolonged physical exercise. NO has a key role in governing oxidative phosphorylation and mitochondrial biogenesis in skeletal muscle^32^. Therefore, the increase of NO production in myotubes cultured in low Mg media might represent a mechanism used by the skeletal muscle cells to adapt to Mg deficiency, modifying their metabolism towards mitochondrial respiration. Coherently, the treatment of myotubes cultured in low Mg with a NOS inhibitor reinstates the LD content, restores the beta-oxidation rate and revitalizes the autophagic flux. It also thwarted the reduction in myotube thickness and the MyHC I (slow) vs. MyHC II (fast) ratio increase.

We are aware of the limitations of using an in vitro model of differentiated C2C12 cells. While these cells express essential muscle proteins and exhibit certain morphological features, they do not fully represent adult muscle due to differences in maturation. Despite this limitation, our in vitro approach remains a valuable tool to understand the intricate interplay between Mg availability, metabolic dynamics and muscle fiber phenotype.

In summary, our results contribute to elucidating the mechanisms underlying the impact of low Mg on skeletal muscle and might have implications for devising strategies to counteract Mg-deficiency related muscle impairment and enhancing overall muscle health and function.

## Methods

### Cell culture

C2C12 murine myoblasts were purchased from American Type Culture Collection (ATCC, St. Louis, Missouri, USA). The cells were serially passaged at 50% confluence in culture medium (CM) composed of DMEM high glucose added with 20% of heat-inactivated fetal bovine serum (FBS), glutamine (2 mM) and 1% penicillin/streptomycin.

To induce myogenic differentiation, cells were seeded at a density of 35,000 cells/cm^2^ and after 24 h of culture in CM, myoblasts were shifted in differentiation medium (DM) consisting of DMEM high glucose added with 2% horse serum.

For all the experiments, myotubes were obtained after 6 days in standard DM and then exposed for 4 days to DM containing severe low (0.1 mM), mild low (0.5 mM) and physiological (1 mM) MgSO_4_ concentrations. 4 days of culture in different Mg concentrations is the optimal timing to avoid detachment of the neo-formed myotubes.

To inhibit nitric oxide (NO) production, myotubes were pre-treated overnight and treated for all the experiment with the generic inhibitor of nitric oxide synthases L-NAME (Sigma-Aldrich, St. Louis, Missouri, USA) at the final concentration of 100 µM. The concentration was defined by performing dose–response MTT assays (data not shown).

Images of cultured cells were acquired with a contrast-phase microscope (Zeiss). Myotubes thickness was measured with the software ImageJ starting from at least 10 random images acquired for each sample. The thickness of each myotube was obtained as the mean of three length measurements in three different positions of the cell.

### SDS–PAGE and western blot

Total protein extracts were obtained with a lysis buffer (50 mM Tris–HCl pH 7.4, 150 mM NaCl, 1% NP-40, 0.25% Na-deoxycholate) added with protease inhibitors (10 µg/mL Leupeptin, 10 µg/mL Aprotinin and 1 mM Phenylmethylsulfonyl fluoride, PMSF) (Sigma-Aldrich, St. Louis, Missouri, USA) and phosphatase inhibitors (1 mM sodium fluoride, 1 mM sodium vanadate, 5 mM sodium phosphate). A syringe was used to obtain a better homogenization of the samples. Total protein extracts were quantified with Bradford assay and 20–30 µg of proteins were separated by SDS-PAGE on Mini-PROTEAN TGX Stain-free Gels (Bio-Rad, Hercules, California, USA) and transferred to nitrocellulose membranes by using Trans-Blot® TurboTM Transfer Pack (Bio-Rad, Hercules, California, USA). After blocking with bovine serum albumin (BSA), western blot analysis was performed using primary antibodies against Myosin Heavy Chain (MyHC) (1:1000) (MAB4470, R&D Systems, Minneapolis, USA), Myog (1:1000) (556358, BD Transduction Laboratories, Milano, Italy), MyoD (1:200) (sc-32758, Santa-Cruz Biotechnology, Dallas, TX, USA), GLUT4 (1:1000) (MA517176, Invitrogen, Thermo Fisher Scientific, Waltham, MA, USA), pAkt (1:2000) (4060 s, Cell Signalling, Danvers, MA, USA), GAPDH (1:200) (sc-25778, Santa-Cruz Biotechnology, Dallas, TX, USA), PLIN2 (1:1000) (AB108323, Abcam, Cambridge, UK), CD36 (1:1000) (PA5-27236, Invitrogen, Thermo Fisher Scientific, Waltham, MA, USA), ATGL (1:1000) (AB109251, Abcam, Cambridge, UK), CPT1A (1:1000) (PA5-29995, Invitrogen, Thermo Fisher Scientific, Waltham, MA, USA), “fast” and “slow” MyHC (1:3000) (m4276 and m8421, Sigma Aldrich, St. Louis, Missouri, USA), LC3B (1:1000) (3868 s, Cell Signalling, Danvers, MA, USA), NOS1 (1:200) (sc-1025 M, Santa-Cruz Biotechnology, Dallas, TX, USA) and Vinculin (1:1000) (AB219649, Abcam, Cambridge, UK).

After extensive washing, nitrocellulose membranes were incubated with secondary antibodies conjugated with horseradish peroxidase (GE Healthcare, Waukesha, WI, USA). The immunoreactive proteins were detected with Clarity™ Western ECL substrate (Bio-Rad, Hercules, California, USA) and images were acquired with a ChemiDoc MP Imaging System (Bio-Rad, Hercules, California, USA). Densitometry of the bands was performed with the software ImageLab (Bio-Rad, Hercules, California, USA). The western blots shown are representative and the densitometric analysis was performed at least on three independent experiments.

### Immunofluorescence

The immunofluorescence staining and imaging were performed directly in culture wells. Cells were seeded in 24-wells plates and, at the end of the experiments, were fixed for 15 min in phosphate-buffered saline (PBS) containing 4% paraformaldehyde and 2% sucrose (pH 7.6). Cells were permeabilized and blocked for 30 min in a PBS solution containing 2% BSA and 0.3% Triton. To stain myotubes, the cells were incubated with anti-MyHC primary antibody (MAB4470, R&D Systems, Minneapolis, USA) and with an Alexa Fluor 488 secondary antibody (A11001, Thermo Fisher Scientific, Waltham, MA, USA). The nuclei were stained with 4′,6-diamidino-2-phenylindole (DAPI). Finally, images were acquired using FLoid™ Cell Imaging Station (Thermo Fisher Scientific, Waltham, MA, USA).

### Lipid droplet staining

Intracellular lipid droplets were stained with the neutral fluorescent probe Bodipy™ (Thermo Fisher Scientific, Waltham, MA, USA). Cells were seeded on 96-well black plate (Costar, Sigma‐Aldrich, St. Louis, MO, USA) and at the end of the experiment they were incubated for 30 min at 37 °C with Bodipy© (λex = 494 nm; λem = 517 nm) and co-stained with Hoechst 33,342 (λex = 361 nm; λem = 497 nm) (Thermo Fisher Scientific, Waltham, MA, USA) for nuclear detection and fluorescence normalization. The fluorescence was acquired at Varioskan LUX Multimode Microplate Reader (Thermo Fisher Scientific, Waltham, MA, USA). The assay was performed at least in triplicate and repeated three times.

For images acquisition, cells were cultured on 24-well plates, co-stained with Bodipy and Hoechst 33,342 and fixed for 15 min in phosphate-buffered saline (PBS) containing 4% paraformaldehyde and 2% sucrose (pH 7.6). Imaged were acquired using FLoid™ Cell Imaging Station (Thermo Fisher Scientific, Waltham, MA, USA).

### Tryglyceride (TG) quantification

TGs were quantified using the Triglyceride Quantification Kit (Sigma-Aldrich, St. Louis, Missouri, USA). Cells were seeded in 6-well plates. At the end of the experiments, cells were detached from the plate and counted. 1 × 10^6^ cells were lysed in 1 ml H_2_O + 5% NP-40 (Sigma-Aldrich, St. Louis, Missouri, USA). Then the samples were hit at 80 °C for 5 min and centrifuged for 2 min at 14,000 rpm to remove the unsoluble material. The samples were aliquoted in a 96-well black plate (Costar, Sigma‐Aldrich, St. Louis, MO, USA) at least in triplicate. Lipase was added to each well and incubated for 20 min at room temperature (RT), so that TGs were broken down into free fatty acids and glycerol. By adding the Assay Reaction Mix and incubating for 1 h RT, glycerol was then oxidized to generate a fluorescent product (λex = 535; λem = 587 nm). The fluorescence was acquired at Varioskan LUX Multimode Microplate Reader (Thermo Fisher Scientific, Waltham, MA, USA). A standard curve was built to calculate the TGs concentration in the samples (ng/µl). The assay was performed in triplicate and repeated three times.

### Autophagic flux analysis

To study autophagy, CYTO‐ID autophagy detection kit (Enzo Life Sciences, Euroclone S.p.A., Pero, Italy) was used. The cells were seeded on a 96‐well black plate (Costar, Sigma‐Aldrich, St. Louis, MO, USA) and at the end of the experiment they were incubated for 30 min at 37 °C with CYTO‐ID detection reagent (λex = 480 nm, λem = 530 nm) for autophagic vesicles staining and co‐stained with Hoechst 33342 (Thermo Fisher Scientific, Waltham, MA, USA) for nuclear detection and fluorescence normalization.

### ROS production analysis

For the detection of ROS, cells were cultured in 96-well black plates (Costar, Sigma‐Aldrich, St. Louis, MO, USA). At the end of the experiment, cells were incubated with 10 mM 2ʹ-7ʹ-dichlorofluorescein diacetate (DCFDA) (Thermo Fisher Scientific, Waltham, MA, USA) solution for 30 min at 37 °C in the dark. DCFDA is deacetylated by cellular esterases to a non-fluorescent compound which is then oxidized by ROS into the fluorescent molecule 2ʹ,7ʹ-dichlorofluorescein (DCF) (λex = 495 nm, λem = 529 nm). DCF fluorescence was normalized on MTT assay performed in parallel.

To measure the amount of mitochondrial ROS, cells were incubated for 10 min at 37 °C with the probe Mitosox (Molecular Probes, Thermo Fisher Scientific, Waltham, MA, USA) (λex = 510 nm, λem = 580 nm) and co‐stained with Hoechst 33,342 for nuclear detection and fluorescence normalization.

### Intracellular lactate quantification

The Lactate-Glo™ assay (Promega, Milan, Italy) couples lactate oxidation and NADH production with a bioluminescent detection system. In particular, Lactate Dehydrogenase uses lactate and NAD + to produce pyruvate ad NADH. In the presence of NADH a pro-luciferin Reductase Substrate is converted by Reductase to luciferin, which is then used in a luciferase reaction to produce light. The cells were seeded in a 96-well white plate (Costar, Sigma‐Aldrich, St. Louis, MO, USA). At the end of the experiment, myotubes were rinsed with ice-cold PBS twice and incubated for 5 min RT with Inactivation Solution (0.6N HCl in water) which stops metabolism and allows cell lysis, deproteinization and the inactivation of NAD(P)H. Then, Neutralization Solution (1 M Trizma®) was added to each well. Lactate Detection Reagent was prepared according to manufacturer’s instructions ad added to each assay well. The plate was incubated for 1 h RT in the dark and luminescence was acquired with Varioskan LUX Multimode Microplate Reader (Thermo Fisher Scientific, Waltham, MA, USA). Data were normalized on cell viability assessed by MTT assay performed in parallel. The assay was performed at least in triplicate and repeated three times.

### Griess assay

The Griess assay measures nitrites (NOx) in the culture medium, which are the oxidation products of nitric oxide. Cells were seeded in 24-well plates and, at the end of the experiment, 500 µl of conditioned media were taken. To remove the protein fraction, culture media were mixed with the same volume of acetone, vortexed very strongly and centrifuged for 10 min at 14,000 rpm at 4 °C. The supernatant was transferred in a new tube and aliquoted in a 96-well plate (100 µl/well) at least in triplicate. Deproteinized fresh culture medium was used as blank. To each well, 100 µl of freshly prepared Griess reagent were added and absorbance was measured at 505 nm after 0, 10, 20, 30 and 40 min. All the measurements were performed at 37 °C. The concentration of nitrites in the samples was determined using a calibration curve generated with serial dilutions of a 100 µM solution of sodium nitrite (NaNO_2_).

### Fatty acid oxidation (FAO) analysis

FAO, the primary metabolic pathway for the degradation of fatty acids, was monitored by Fatty Acid Oxidation assay (Abcam, Cambridge, UK) in living myotubes. The cells were seeded in a 96-well black plate (Costar, Sigma‐Aldrich, St. Louis, MO, USA). At the end of the experiment, cells were incubated overnight with glucose-free medium added with L-carnitine (0.5 mM) (Abcam, Cambridge, UK). For the assay, cells were rinsed twice with pre-warmed Fatty Acid-Free medium added with 0.5 mM L-carnitine and 2.5 mM D-Glucose. Then pre-warmed Fatty Acid Measurement Medium, prepared by adding Oleate (FAO-conjugate) to Fatty Acid-Free medium, was added to each well. To measure FAO-related oxygen consumption, Extracellular O_2_ Consumption Reagent (Abcam, Cambridge, UK) was added into all the wells except for the blank control well. Carbonyl cyanide-p-trifluoromethoxyphenylhydrazone (FCCP, 0.625 µM), which increases cellular energy demand by inducing maximal electron transport chain activity, was used as the positive control. At the end of the experiment the wells were sealed with pre-warmed high sensitivity mineral oil (Abcam, Cambridge, UK). For the measurement, the 96-well black plate was placed into the Varioskan LUX Multimode Microplate Reader (Thermo Fisher Scientific, Waltham, MA, USA) pre-set to 37 °C. The fluorescent signal (λex = 380; λem = 650 nm) was measured every 2 min for 90 min^33,34^. The results are both expressed as a time-course curve and as a box plot graph. The experiment was performed three times in triplicate.

### Statistical analysis

Data are expressed as the mean ± standard deviation (SD). Normal distribution of data sets was evaluated with D’Agostino-Pearson normality test. In the case of data not normally distributed, statistical significance was calculated with non-parametric one-way ANOVA (Kruskal–Wallis test) and the p-values were corrected using the Dunn’s method.

In the case of normally distributed data set, parametric one-way ANOVA was used and the p-values were corrected using the post-hoc Tukey’s method. In all the figures, *p ≤ 0.05; **p ≤ 0.01; ***p ≤ 0.001. All statistical analyses were performed with the software GraphPad Prism.

### Supplementary Information

Supplementary Figures.

## Data Availability

The data presented in this study are openly available in Dataverse at https://dataverse.unimi.it/dataverse/myotubes_and_low_Mg.
