# Supplementary material for: Low extracellular magnesium induces phenotypic and metabolic alterations in C2C12-derived myotubes
Source: Sci Rep. 2023 Nov 8;13:19425. doi: 10.1038/s41598-023-46543-9 (PMC10632379; doi:10.1038/s41598-023-46543-9)
Supplement: Supplementary file 1 — Supplementary Figures. [file 41598_2023_46543_MOESM1_ESM.pdf]

Low extracellular Mg induces phenotypic and metabolic alterations in C2C12-derived myotubes

Monica Zocchi, Jeanette A. Maier and Sara Castiglioni*

Department of Biomedical and Clinical Sciences, Università di Milano, 20157 Milano, Italy.

*sara.castiglioni@unimi.it

Supplementary information

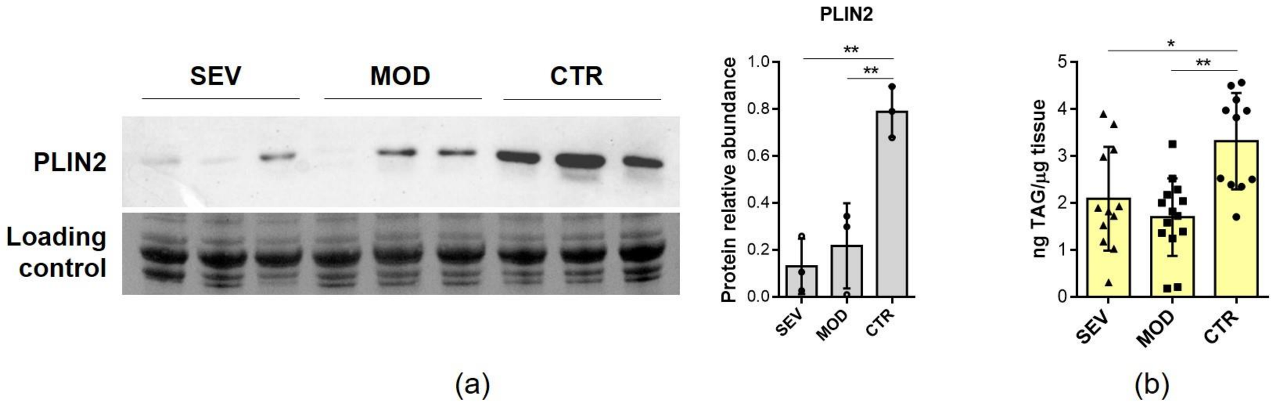

Fig. S1 Mg deficiency affects lipid metabolism *in vivo*. Mice were fed either a control (CTR, 0.1% Mg wt/wt), a mildly (MOD, 0.01% Mg wt/wt) or a severely (SEV, 0.003% Mg wt/wt) Mg-deficient diet for 14 days. (a) PLIN2 expression in gastrocnemius skeletal muscle was analyzed by western blot. A representative blot (left) and densitometry obtained by ImageLab (right) are shown. (b) TGs content was measured in the gastrocnemius skeletal muscle as described in Materials and Methods. * $p \leq 0.05$, ** $p \leq 0.01$.

Uncropped western blots

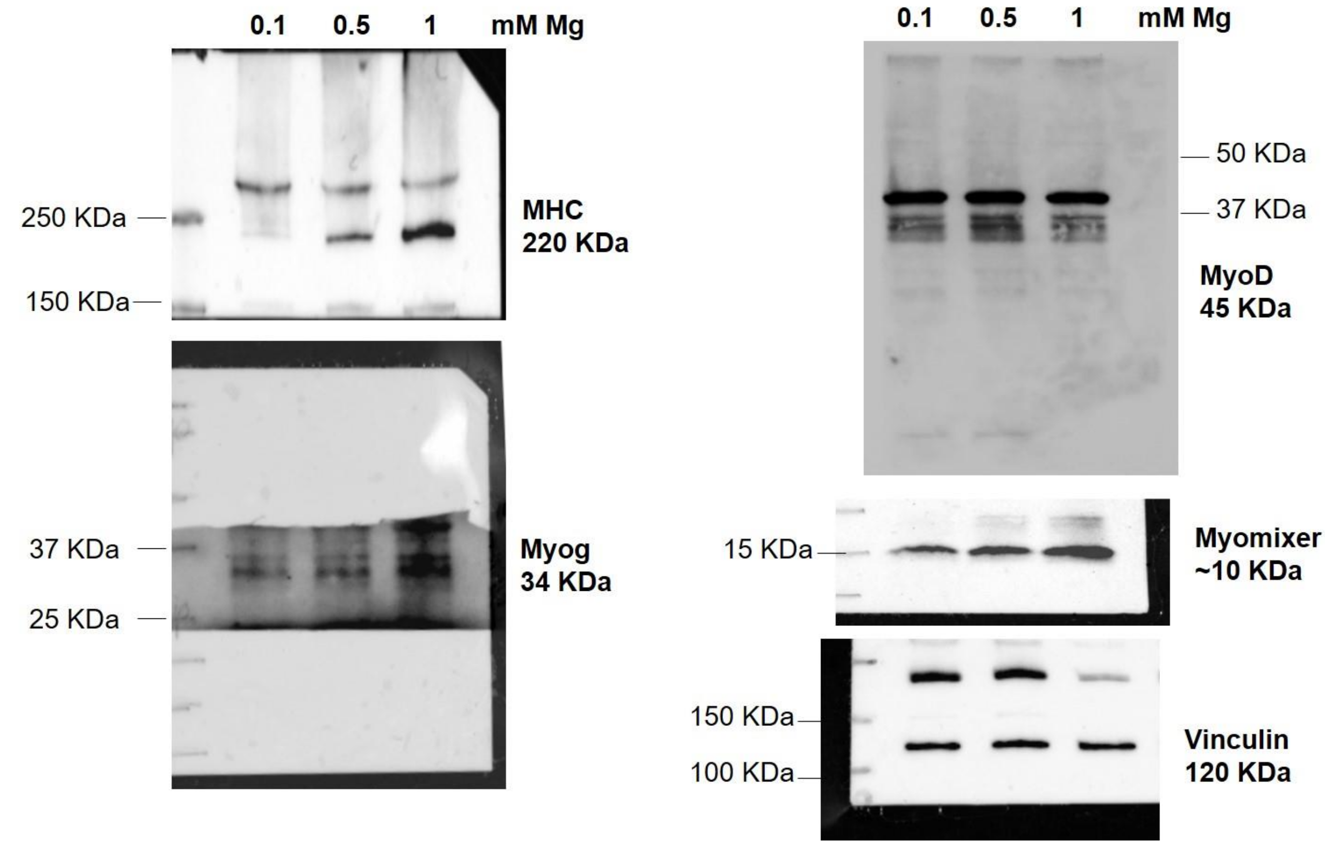

Fig. 1

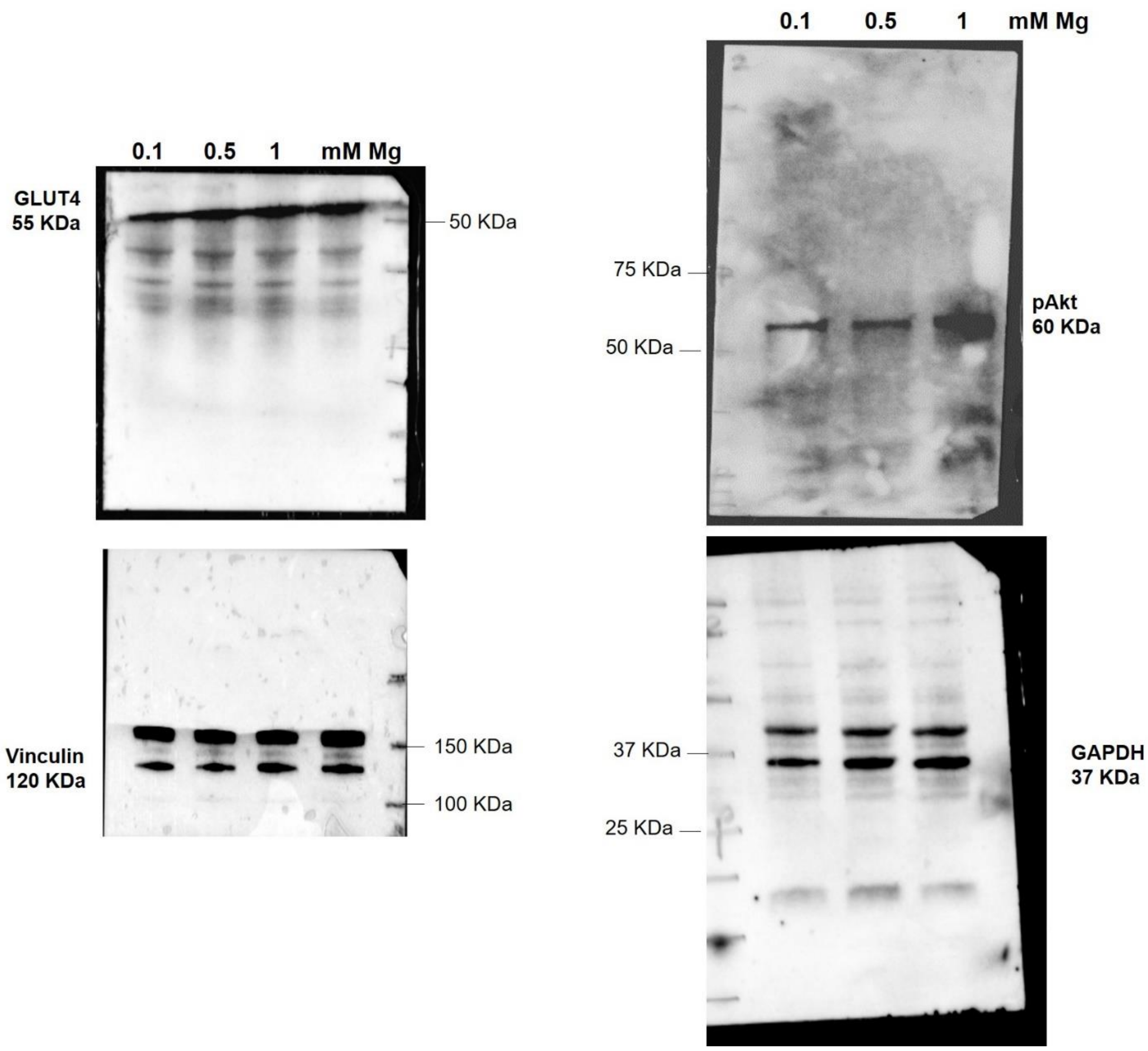
0.1
0.5
1
mM Mg
GLUT4
55 KDa
50 KDa
Vinculin
120 KDa
150 KDa
100 KDa
0.1
0.5
1
mM Mg
75 KDa
50 KDa
pAkt
60 KDa
37 KDa
25 KDa
GAPDH
37 KDa

Fig. 2

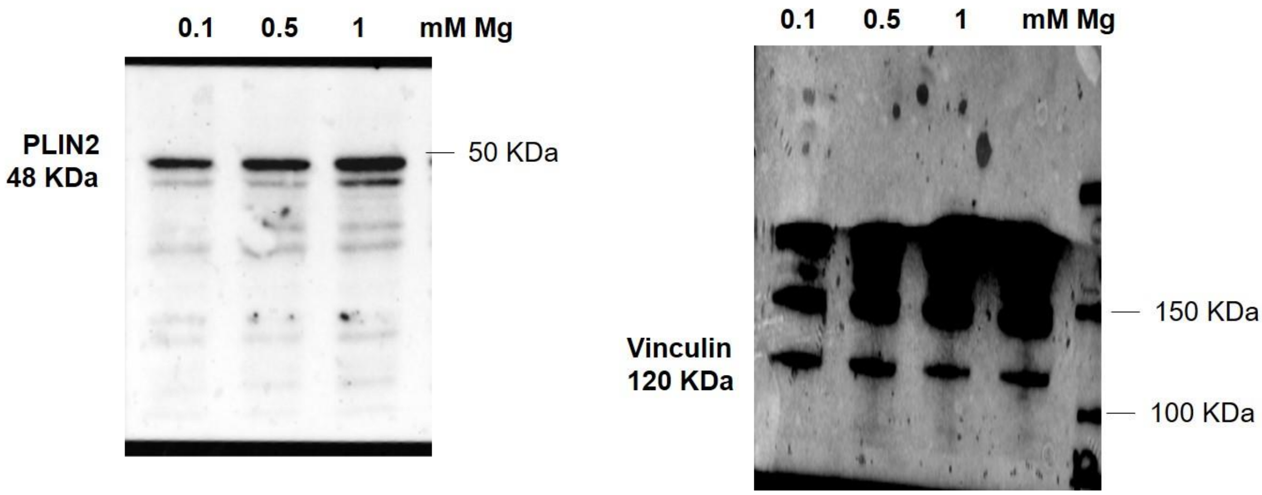
0.1
0.5
1
mM Mg
PLIN2
48 KDa
50 KDa
0.1
0.5
1
mM Mg
Vinculin
120 KDa
150 KDa
100 KDa

Fig. 3

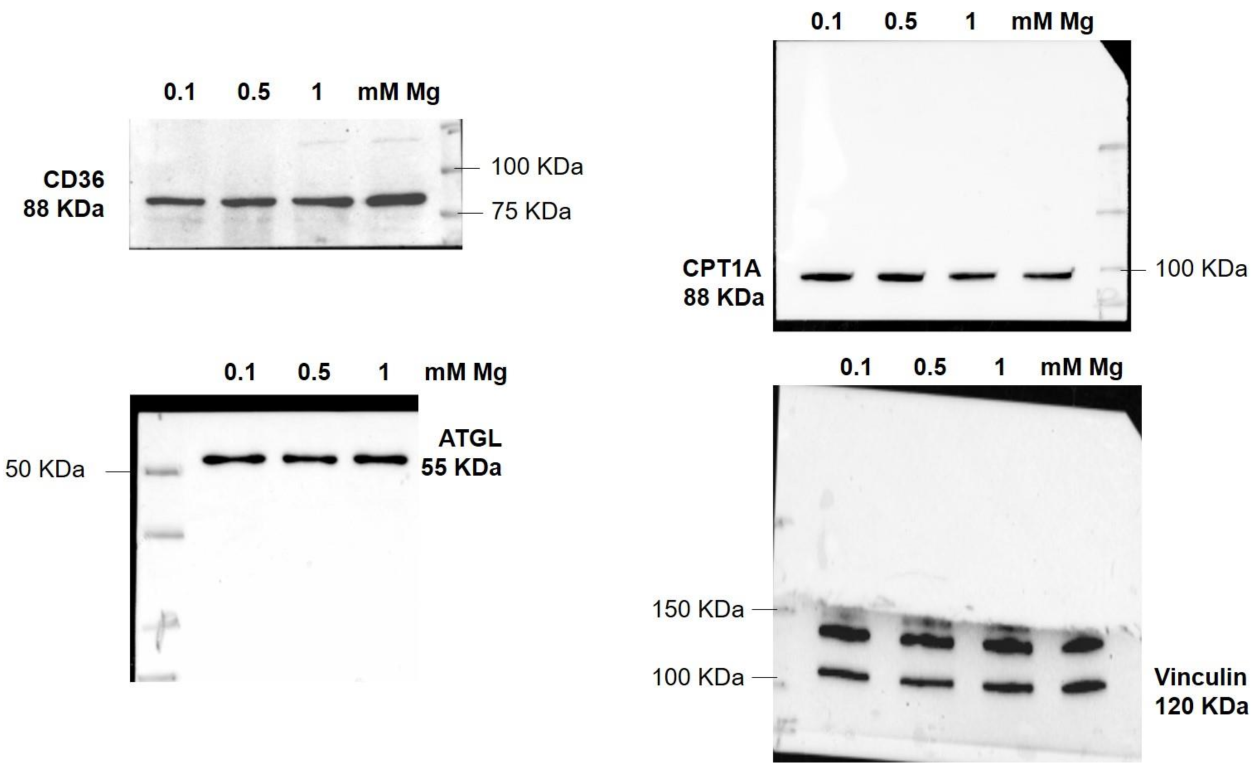
0.1 0.5 1 mM Mg
CD36
88 KDa
100 KDa
75 KDa
0.1 0.5 1 mM Mg
CPT1A
88 KDa
100 KDa
0.1 0.5 1 mM Mg
ATGL
55 KDa
50 KDa
0.1 0.5 1 mM Mg
150 KDa
100 KDa
Vinculin
120 KDa

Fig. 4

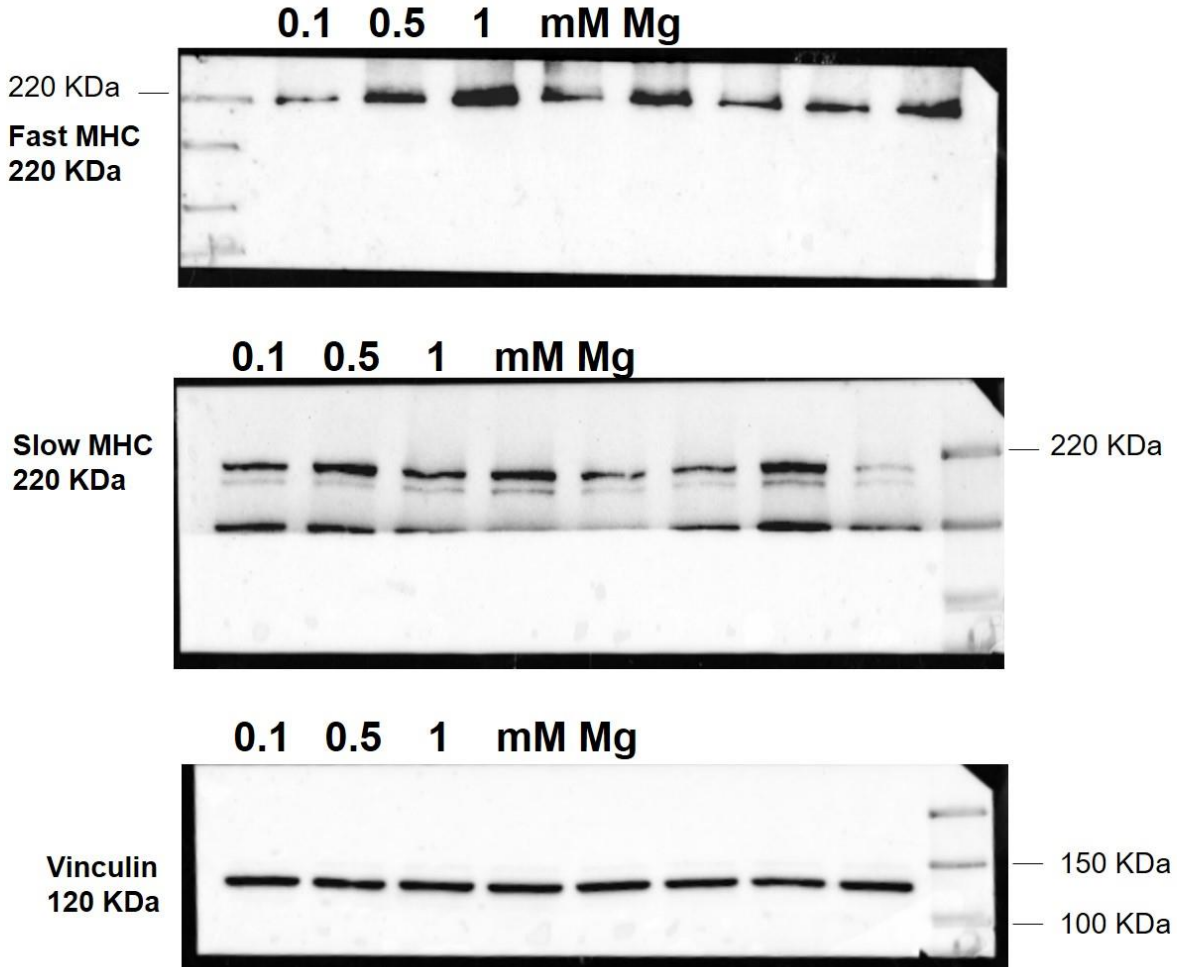
0.1 0.5 1 mM Mg
220 KDa
Fast MHC
220 KDa
0.1 0.5 1 mM Mg
Slow MHC
220 KDa
220 KDa
0.1 0.5 1 mM Mg
Vinculin
120 KDa
150 KDa
100 KDa

Fig. 5

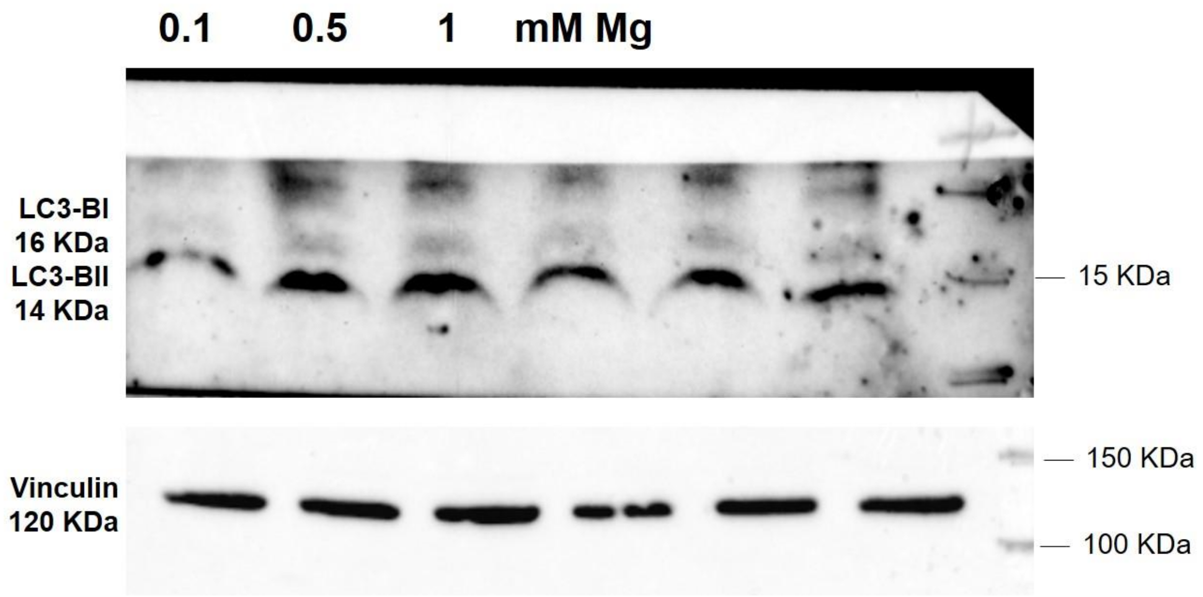
0.1
0.5
1
mM Mg
LC3-BI
16 KDa
LC3-BII
14 KDa
15 KDa
Vinculin
120 KDa
150 KDa
100 KDa

Fig. 6

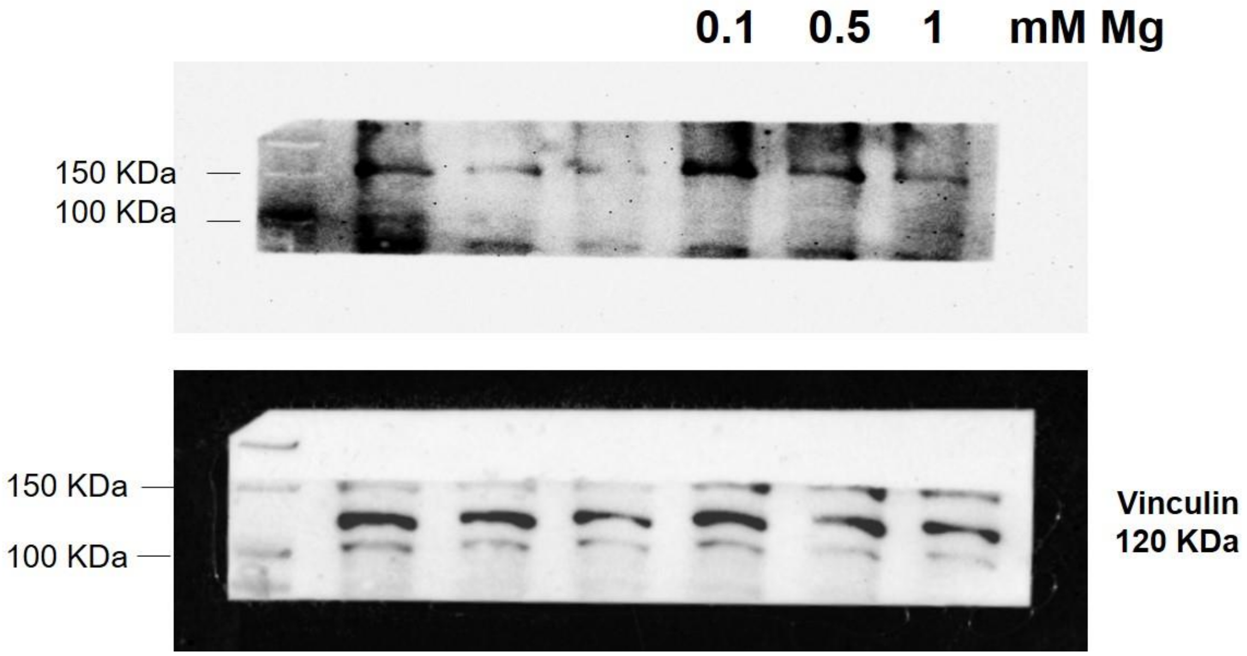
0.1
0.5
1
mM Mg
150 KDa
100 KDa
150 KDa
100 KDa
Vinculin
120 KDa

Fig. 7
